# Analysis of the mortality according to reperfusion therapy used in the acute coronary syndrome

**DOI:** 10.1186/2197-425X-3-S1-A752

**Published:** 2015-10-01

**Authors:** L Navarro Guillamón, MR Díaz Contreras, V Chica Saénz, E Aguayo de Hoyos, A Reina Toral, Group ARIAM

**Affiliations:** H.U. Virgen de las Nieves, Granada, Spain

## Objective

To evaluate the results of the fibrinolysis vs ICP 1st in our province as a response to the initiative of the Provincial Bureau of reperfusion. In many centers the ICP 1st is not an option, not having any service of hemodynamics and referral to a center if available can be a delay not affordable.

## MATERIAL AND METHODS

A retrospective study of patients admitted to the ICU with a diagnosis of acute coronary syndrome during a period of 4.5 years (1-Jan-2009 to 30-June-2013) in the province of Granada. We will use the database of the register of ischemic heart disease in Andalusia (group ARIAM). The statistical analysis was performed with a logistic regression model taking as binary dependent mortality in the ICU and adjusting the strategy of reperfusion, age, sex and Killip initial. After fits a logistic regression model with the polynomial of reperfusion strategy as dependent and explanatory mortality, age and initial Killip to study if there are differences between ICP 1st and early fibrinolysis.

## Results

The total number of patients admitted to the ICU with a diagnosis of STEMI in that period was 1767. The 77.8% were male and 22.2% female. The average age was 62.13 and 70.3 respectively. In Killip III-IV admitted 10% of patients.

Reperfusion strategy used has been divided into none (N), primary PCI (IP), early fibrinolysis (EF) and late fibrinolysis (LF), these last two distinct if committed before or after three hours of onset of pain. In each year have made respectively:

2009: 81 N, the IP 57 and 149 EF and 99 FT.

2010: 78 N, 61 IP, EF 148 and 79 LF.

2011: 97N, 91 IP, EF 133 and 104 LF.

2012: 96N, 104 IP, 130 EF and 80 LF.

2013 (until June 30): 39 N, 61 IP, 47 EF and 42 LF.

The overall mortality by reperfusion technique used was 4% when none was used, 6% in ICP 1, 6% in early fibrinolysis and 9% late fibrinolysis.

By adjusting the binary logistic regression model we obtain that none of the techniques used is related to mortality. Yes they do age and Killip on admission.

In the polytomous logistic regression model to note that early fibrinolysis and PCI draws on 1st does not show a statistically significant mortality outcome variable.

## Conclusions

The results in our province in terms of mortality in ICU between primary PCI and fibrinolysis early show no statistically significant difference.

